# The Danger Signal Extracellular ATP Is an Inducer of *Fusobacterium nucleatum* Biofilm Dispersal

**DOI:** 10.3389/fcimb.2016.00155

**Published:** 2016-11-17

**Authors:** Qinfeng Ding, Kai Soo Tan

**Affiliations:** Faculty of Dentistry, National University of SingaporeSingapore, Singapore

**Keywords:** eATP, *Fusobacterium nucleatum*, FadA, bacterial invasion, inflammation, DAMP, periodontal disease

## Abstract

Plaque biofilm is the primary etiological agent of periodontal disease. Biofilm formation progresses through multiple developmental stages beginning with bacterial attachment to a surface, followed by development of microcolonies and finally detachment and dispersal from a mature biofilm as free planktonic bacteria. Tissue damage arising from inflammatory response to biofilm is one of the hallmark features of periodontal disease. A consequence of tissue damage is the release of ATP from within the cell into the extracellular space. Extracellular ATP (eATP) is an example of a danger associated molecular pattern (DAMP) employed by mammalian cells to elicit inflammatory and damage healing responses. Although, the roles of eATP as a signaling molecule in multi-cellular organisms have been relatively well studied, exogenous ATP also influences bacteria biofilm formation. Since plaque biofilms are continuously exposed to various stresses including exposure to the host damage factors such as eATP, we hypothesized that eATP, in addition to eliciting inflammation could potentially influence the biofilm lifecycle of periodontal associated bacteria. We found that eATP rather than nutritional factors or oxidative stress induced dispersal of *Fusobacterium nucleatum*, an organism associated with periodontal disease. eATP induced biofilm dispersal through chelating metal ions present in biofilm. Dispersed *F. nucleatum* biofilm, regardless of natural or induced dispersal by exogenous ATP, were more adhesive and invasive compared to planktonic or biofilm counterparts, and correspondingly activated significantly more pro-inflammatory cytokine production in infected periodontal fibroblasts. Dispersed *F. nucleatum* also showed higher expression of *fadA*, a virulence factor implicated in adhesion and invasion, compared to planktonic or biofilm bacteria. This study revealed for the first time that periodontal bacterium is capable of co-opting eATP, a host danger signaling molecule to detach from biofilms. Our results further showed that dispersed *F. nucleatum* possessed distinct virulence characteristics compared to their biofilm and planktonic counterparts.

## Introduction

Plaque biofilm is the primary etiological agent of periodontal disease which is the main cause of tooth loss in adults (Marsh, 1994, 2003). Biofilms consist of bacterial communities embedded in extracellular polymeric substances (EPS) composed of polysaccharides, proteins, and extracellular DNA (Costerton et al., 1995). Biofilm formation typically progresses through multiple developmental stages beginning with bacterial attachment to a surface, followed by development of microcolonies. A challenge in eradicating bacteria in biofilm is their increased resistance to antimicrobial agents and host defenses compared to their planktonic counterparts (Mah and O'toole, 2001). The final stage of biofilm development is the detachment of cells where bacteria disperse from a mature biofilm as free planktonic bacteria. Dispersal is an important stage in the biofilm life cycle that contributes to bacterial survival, persistence, and disease transmission (Kaplan, 2010). Dispersed bacteria have been reported to be physiologically distinct from their sessile counterparts, exhibiting different gene expression profiles (Sauer et al., 2002). Furthermore, dispersed *Pseudomonas aeruginosa* have been found to possess enhanced virulence compared to planktonic cells (Chua et al., 2014).

Dispersal of biofilm is a complex process and can be enhanced or repressed in response to diverse environmental cues such as changes in nutrients, oxygen tension, pH, exposure to EPS degrading enzymes such as dispersin B, or signaling molecules such as nitric oxide or cis-2-decenoic acid (Kaplan et al., 2003; Barraud et al., 2006; Davies and Marques, 2009; Karatan and Watnick, 2009; Kaplan, 2010). There is no universal mechanism of biofilm dispersal which is conserved across all bacteria. For instance, increased availability of carbon substrates induces dispersal of *P. aeruginosa* while starvation and stress induces dispersal of *P. fluorescens* and *P. putida* (Delaquis et al., 1989; Gjermansen et al., 2010). Beside environmental factors, molecules which are released from stressed or damaged host cells such as adenosine triphosphate (ATP) also stimulate adhesion and biofilm formation of nosocomial pathogens (Xi and Wu, 2010).

Adenosine triphosphate (ATP) is the principal energy currency used by living organisms for cellular metabolism within the cell. However, stressed or damaged host cells secrete ATP into the extracellular environment. A rapid increase of ATP concentration in the extracellular environment is a danger signal which alerts immune cells of an impending threat, and mobilizes a rapid inflammatory response to clear invading pathogens. Exacerbated host inflammatory response against plaque bacteria lead to hallmark features of periodontal disease including gingival inflammation, pocket formation, tissue damage, and alveolar bone loss (Page et al., 1978). Presence of ATP in the extracellular environment has been shown to exacerbate periodontitis. For instance, Binderman et al. reported that by decreasing the levels of extracellular ATP (eATP) through application of ATP hydrolyase or applying antagonists to block eATP-mediated activation of purinergic receptors significantly reduced alveolar bone loss in periodontitis (Binderman et al., 2000). ATP also activates osteoclasts on alveolar bone surfaces during periodontitis in marginal gingival cells leading to bone destruction (Binderman et al., 2007). In addition, exogenously added ATP also causes growth arrest and influences periodontal tissue regeneration (Kawase et al., 2007).

Bacteria implicated in periodontal disease are predominantly strict anaerobes such as *Fusobacterium nucleatum, Porphyromonas gingivalis, Treponema denticola*, and *Tannerella forsythia* (Socransky et al., 1998). *F. nucleatum* is a gram negative spindle shaped anaerobe commonly found in plaque biofilm. In plaque biofilm, *F. nucleatum* plays an important role as a bridging organism to adhere to early and late dental plaque colonizers and stabilize the developing plaque biofilm (Bradshaw et al., 1998). In the absence of *F. nucleatum* the amount of the late colonizers associated with periodontal destruction is significantly reduced. This organism has also been detected with higher prevalence in patients with increasing probing depths (Haffajee et al., 2006).

In the subgingival environment where these periodontal bacteria reside, these organisms are subjected to changes in nutrient availability and oxidative stress as a consequence of mechanical plaque disruption, and host damage factors such as eATP. However, it is unknown if these factors influence biofilm dispersal of periodontal bacteria, and if dispersed versus biofilm forms have distinct capacity to elicit inflammation. Given the essential role of *F. nucleatum* in plaque biofilm, this study aims to determine if nutritional factors, oxidative stress and eATP modulate biofilm dispersal of *F. nucleatum*, and to determine if dispersed bacteria differ in its capacity to elicit inflammation compared to planktonic or biofilm forms of *F. nucleatum*.

## Materials and methods

### Bacterial culture

*F. nucleatum* ATCC 23726 was obtained from the American Type Culture Collection, USA. *F. nucleatum* was cultured in brain heart infusion (BHI) broth (Acumedia, USA) supplemented with 0.5% yeast extract (Acumedia), 5 μg/mL hemin and 1 μg/mL vitamin K (Sigma-Aldrich, USA), and incubated at 37°C in an anaerobic chamber supplemented with 80% N_2_, 10% H_2_, and 10% CO_2_ (Don Whitley Scientific, USA).

### Biofilm dispersal assay

Biofilm dispersal assay was performed as described previously (Davies and Marques, 2009). *F. nucleatum* biofilm was cultured in a 96-well flat bottom plate (ThermoFisher Scientific, USA) by diluting an overnight bacterial culture 1:20. Biofilms were incubated at 37°C in an anaerobic workstation (Don Whitley Scientific). Culture media was changed daily. The ability of either 2 mM D-Glucose (Sigma), 20 mM NH_4_Cl (Sigma), 8 mM L-cysteine (Sigma), or 1 mM ATP (Sigma) to induce biofilm dispersal was determined by adding the test compound to 4 day old biofilm in 200 μL BHI and incubated anaerobically for 1 h. Nutrient deprivation was induced by exposing biofilm to PBS. Oxidative stress was induced by incubating *F. nucleatum* aerobically for 1 h at 37°C. The amount of dispersed bacteria was determined by measuring optical density of the culture supernatant at 600 nm. Crystal violet staining was performed to quantify biofilm biomass. Biofilm was washed twice with 1 × PBS to remove planktonic bacteria. Biofilm was fixed by addition of 100% methanol (Sigma) for 10 min and air dried. Subsequently, biofilm was stained with 0.5% crystal violet (Sigma) for 5 min. Unbound crystal violet stain was removed by washing with tap water 5 times and air dried. The wells were filled with 200 μL of 33% acetic acid (v/v) to dissolve crystal violet. Optical density at 580 nm was measured using a plate reader.

### Cell culture

Primary periodontal fibroblasts were purchased from ScienCell, USA. Cells were cultured in DMEM (Hyclone, USA) supplemented with 10% heat-inactivated FBS (Hyclone), 2 mM L-glutamine (Life Technologies, USA), and incubated in a humidified atmosphere with 5% CO_2_ at 37°C.

### *F. nucleatum* adherence and invasion assays

Adherence and invasion assays were carried out as described previously (Han et al., 2000; Lee and Tan, 2014). Periodontal fibroblasts were seeded at a density of 1 × 10^4^ cells into 96-well tissue culture plates (ThermoFisher Scientific). The next day, periodontal fibroblasts were infected with either planktonic, biofilm, or dispersed *F. nucleatum* at a multiplicity of infection (MOI) of 100:1 (bacteria:cells). For infection of periodontal fibroblasts, planktonic bacteria were obtained by serially sub-culturing an overnight culture of *F. nucleatum* at 1:20 for 4 days. *F. nucleatum* biofilm was cultured as described above. Biofilm cells were harvested by vigorous pipetting such that no clumps or aggregates were visible. The disruption of biofilm into single cells was further verified under the microscope. For adherence assays, *F. nucleatum* was incubated with periodontal fibroblasts for 1 h, after which cells were washed with PBS to remove non adherent bacteria. Total cell-associated bacteria were enumerated on serial dilution and plating on TSA supplemented with 5% sheep blood (ThermoFisher Scientific). For invasion assays, periodontal fibroblasts were infected with *F. nucleatum* for 2 h, following which cells were washed with sterile PBS, and incubated for an additional 1.5 h with fresh DMEM (Hyclone) supplemented with 1% FBS (Hyclone) containing gentamicin (Sigma) (200 μg/mL) and metronidazole (250 μg/mL) (Sigma). Cells were lysed with sterile water and the number of intracellular bacteria were determined by serial dilution and plating as described above.

### RNA extraction and qPCR

Following induction of dispersal, dispersed cells were collected and total RNA was extracted with the GeneAll Hybrid-R purification kit (GeneAll Biotechnology, South Korea) according to the manufacturer's protocol. Extracted RNA was treated with RQ1 DNaseI (Promega, USA) to eliminate residual DNA. Reverse transcription was performed using iScript Reverse transcription Supermix (BioRad, USA). mRNA expression of *fadA* and *fap2* genes were determined by quantitative polymerase chain reaction (qPCR) using iTaq Universal SYBR Green Supermix (BioRad) in an Applied Biosystem StepOne Plus. Sequences of the primers used were FadA forward 5′-TGCAGCAAGTTTAGTAGGTG and reverse 5′- CATTGTAAACTTGTTCATTTTGT; Fap2 forward 5′- AAAATTGGAGCAACAGGAGGA and reverse 5′- TTCAGAGGCAATAGCGACAAC; housekeeping gene 16S rRNA forward 5′-CTAAATACGTGCCAGCAGC and reverse 5′-CGACCCCCAACACCTAGTA. Expression of *fadA* and *fap2* was normalized to the relative abundance of 16S rRNA. Fold change was determined by the ΔΔCt method (Livak and Schmittgen, 2001).

### Inhibition of biofilm dispersion

*F. nucleatum* biofilm was cultured as described above. ATP (Sigma) was added with or without 1 mM CaCl_2_ or MgCl_2_ and incubated anaerobically for 1 h. Dispersion of bacteria was determined as described above.

### Enzyme-linked immunosorbent assay (ELISA)

Periodontal fibroblasts were seeded at a density of 1 × 10^4^ cells in a 96-well tissue culture plate and allowed to adhere overnight. Cells were infected with either planktonic, biofilm, or dispersed *F. nucleatum* at a MOI of 100:1 (bacteria:cells) for 4 h. Cell culture supernatant was harvested and the amount of IL-6 and IL-8 present were determined by ELISA kits (catalog number 430,505 and 431,505, respectively) from Biolegend (USA). ELISA was carried out according to the manufacturer's protocol.

### Confocal laser scanning microscopy (CLSM)

*F. nucleatum* biofilm was grown on coverslips in 24 well plates (ThermoFisher Scientific) for 4 days and incubated as described above. Media was changed daily. On the 4th day, biofilm was left untreated or treated with ATP for 1 h and stained with SYTO 9 (Life Technologies). Images were captured with OLYMPUS FLOWVIEW 1000 CLSM (Olympus America, USA).

### Statistical analysis

Results were presented as mean ± SD. Statistical significance was determined by Student's *t*-test or one-way ANOVA with Tukey *post-hoc* analysis using Graphpad Prism software 6.0. Differences were considered significant if *p* < 0.05.

## Results

### ATP induces dispersal of *F. nucleatum*

**Figure 1:**
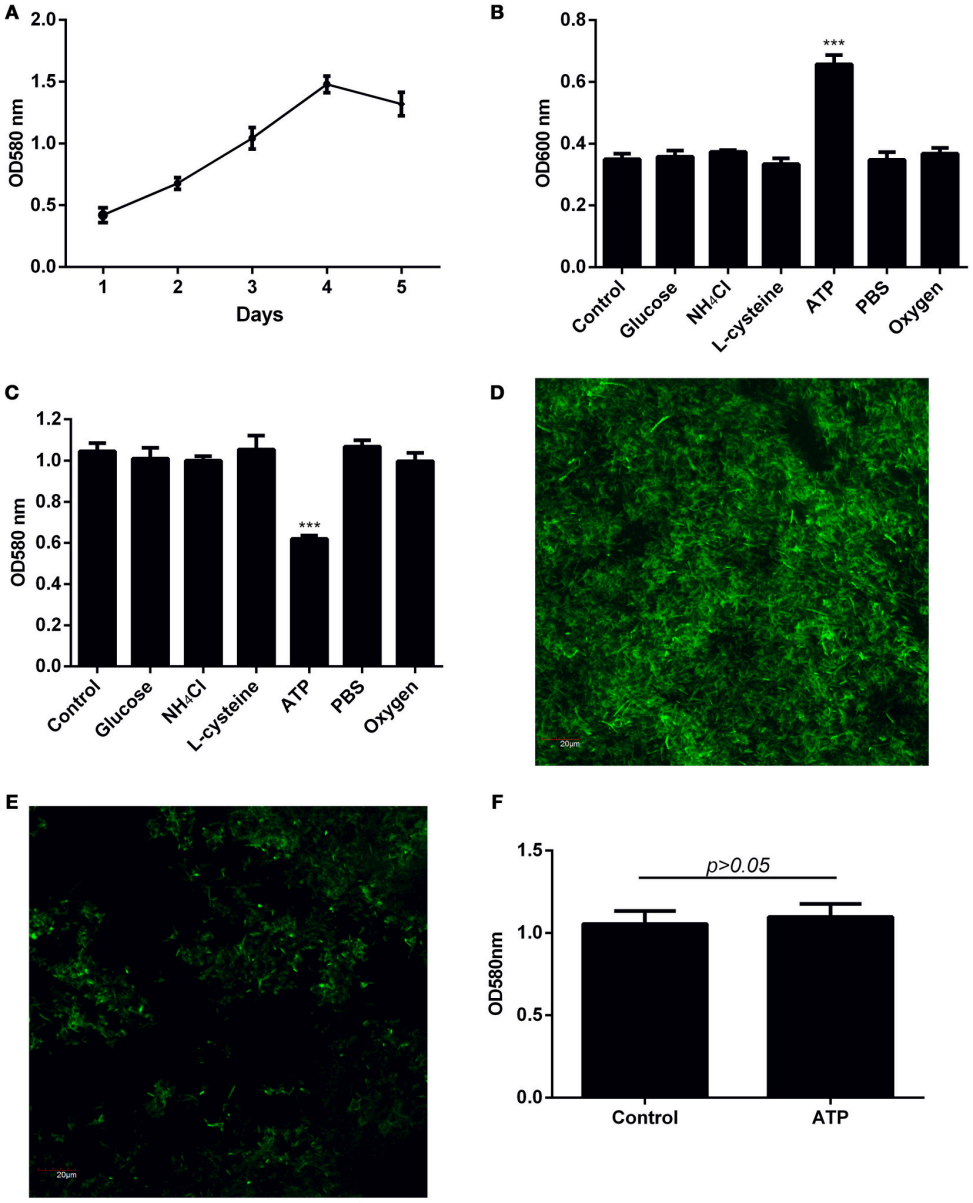
**Investigation of factors inducing dispersal of ***F. nucleatum*** biofilm. (A)** The amount of biofilm formed over a 5-day period was quantified by crystal violet assay. **(B)** Four-day old *F. nucleatum* biofilm was left untreated (control) or treated with 2 mM D-glucose, 20 mM NH_4_Cl, 8 mM L-cysteine, 1 mM ATP or PBS, and incubated anaerobically at 37°C for 1 h. A separate plate of 4-day old *F. nucleatum* biofilm was incubated at 37°C for 1 h aerobically (~20% atmospheric O_2_). The amount of dispersed bacteria was determined by measuring optical density of bacteria in the culture supernatant. **(C)** The amount of biofilm biomass after induction of dispersal was quantified by crystal violet assay. Biofilm grown on cover slips in 24 well plates was left **(D)** untreated or **(E)** treated with ATP for 1 h. The amount of remaining biofilm was visualized by CLSM following staining with SYTO 9. Magnification 60X, scale bar: 20 μm. **(F)** *F. nucleatum* biofilm was cultured in the presence of 1 mM ATP for 4 days with daily change of media. The amount of biofilm formed was quantified by crystal violet assay. The data presented were obtained from one representative experiment carried out in triplicate. All experiments were performed 3 independent times and showed similar trends (Supplementary Figure 1). ^***^p < 0.001.

The amount of *F. nucleatum* biofilm formed was determined over a 5-day period to determine the time required for *F. nucleatum* biofilm to reach climax. Under the culture conditions employed, the biomass peaked at day 4 of culture (Figure 1A, Supplementary Figures 1A,B). Bacteria naturally disperse from biofilm matrix as free planktonic bacteria at low levels in the absence of stimulus. However, dispersion is enhanced in response to certain mediators (Kaplan et al., 2003; Barraud et al., 2006; Davies and Marques, 2009; Gjermansen et al., 2010). To elucidate factors that mediate dispersal of *F. nucleatum* biofilm, dispersal was induced by sudden increase in carbon, amino acid, and nitrogen concentrations in the growth media, depletion of nutrients, or exposure to oxidative stress. Increase availability of either D-glucose, L-cysteine or NH_4_Cl did not significantly induce dispersal of *F. nucleatum* (Figure 1B, Supplementary Figures 1C,D). Similarly, oxidative stress and nutrient deprivation did not impact dispersal (Figure 1B, Supplementary Figures 1C,D). Exposure of established *F. nucleatum* biofilm to ATP concentrations found at sites of tissue damage (Huang et al., 2010; Kitagawa et al., 2013) led to an approximate two-fold increase in dispersal (Figure 1B, Supplementary Figures 1C,D). A corresponding reduction in biofilm biomass was observed after ATP treatment (Figures 1C–E, Supplementary Figures 1E,F). In contrast to established *F. nucleatum* biofilm, addition of ATP during formation of biofilm did not significantly affect the eventual biofilm biomass formed (Figure 1F, Supplementary Figures 1G,H).

### ATP induces biofilm dispersal through metal chelation

**Figure 2:**
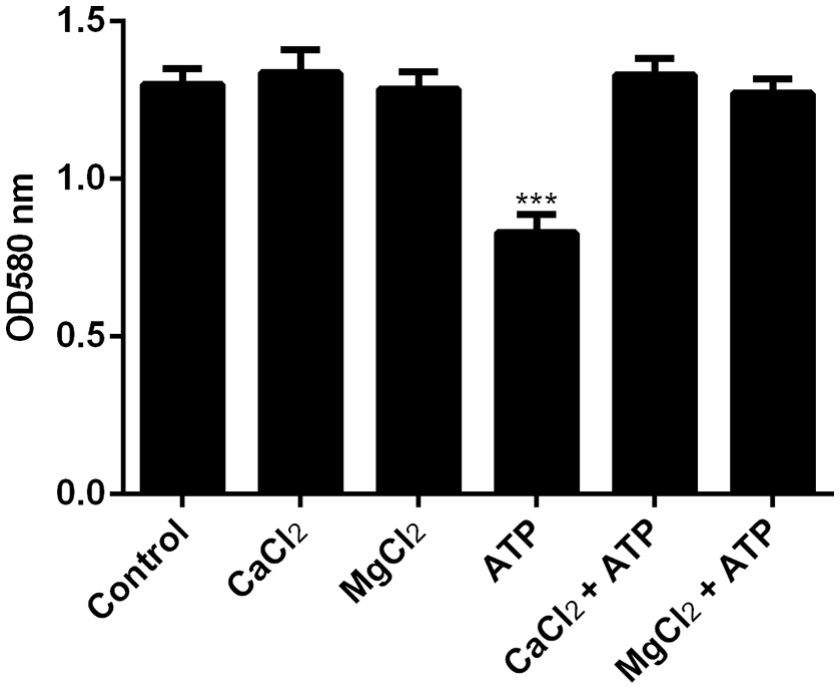
**The effects of cations on ATP-mediated dispersal of biofilm.** *F. nucleatum* biofilm was left untreated (control) or treated with MgCl_2_ or CaCl_2_ in the presence or absence of 1 mM ATP. Biofilm was incubated anaerobically at 37°C for 1 h. The amount of biofilm biomass after induction of dispersal was determined by crystal violet assay. The data presented were obtained from one representative experiment carried out in triplicate. All experiments were performed 3 independent times and showed similar trends (Supplementary Figure 2). ^***^p < 0.001.

ATP has been reported to chelate metal ions such as Mg^2+^ and Ca^2+^ which are essential to maintain the integrity of the EPS (Tatano et al., 2015). To determine if ATP is inducing dispersal by depriving bacterial biofilm of essential metal ions, biofilms were pre-treated with either Mg^2+^ or Ca^2+^ prior to stimulation with ATP. Indeed, pre-treatment with cations obliterated ATP induced bacterial dispersal (Figure 2, Supplementary Figure 2).

### Planktonic, biofilm, and dispersed cells differ in virulence

**Figure 3:**
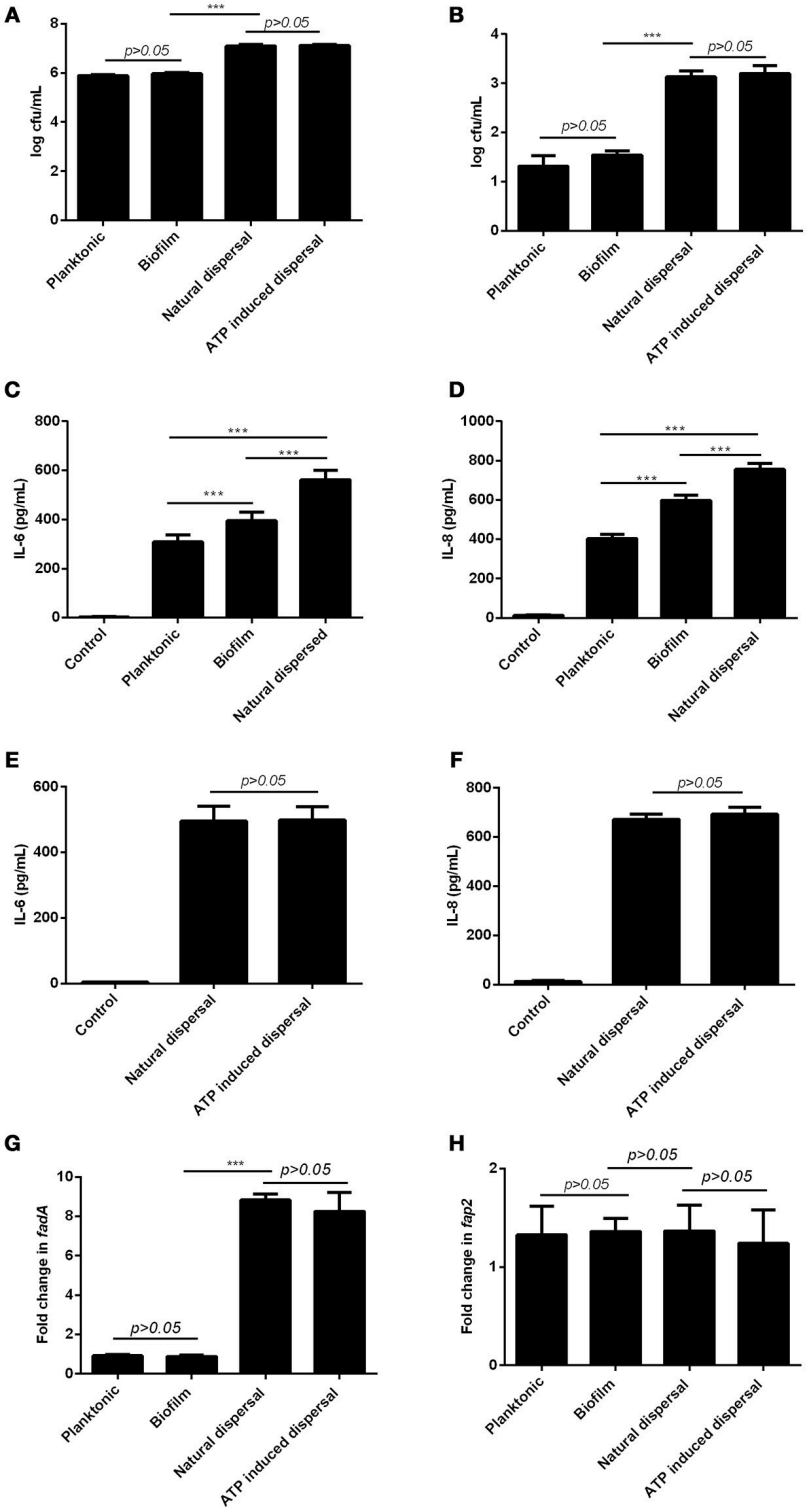
**Differential effects of planktonic, biofilm and dispersed ***F. nucleatum*** on periodontal fibroblasts.** Primary periodontal fibroblasts were infected with planktonic, biofilm, naturally dispersed or ATP induced *F. nucleatum* for 1 h after which **(A)** cell-associated bacteria and **(B)** intracellular bacteria were determined by serial dilution and plating. Periodontal fibroblasts were infected with planktonic, biofilm, naturally dispersed or ATP induced *F. nucleatum* for 4 h and the amounts of **(C,E)** IL-6 and **(D,F)** IL-8 produced were quantified by ELISA. The expression of **(G)** *fadA* and **(H)** *fap2* in planktonic, biofilm and dispersed *F. nucleatum* were determined by qPCR. ^***^p < 0.001.

Since bacterial invasion has been suggested to be a potential pathogenic mechanism for periodontitis (Listgarten, 1986), the ability of planktonic, biofilm, and dispersed *F. nucleatum* to adhere and invade primary periodontal fibroblasts were examined. Planktonic and biofilm forms of *F. nucleatum* were comparable in their ability to adhere and invade primary periodontal fibroblasts (Figures 3A,B). Both naturally dispersed and ATP-induced dispersed *F. nucleatum* biofilm showed significantly increased adhesive and invasive potential (69- and 16-fold increase, respectively) compared to planktonic and biofilm bacteria (Figures 3A,B). Periodontal fibroblasts infected with biofilm *F. nucleatum* elicited significantly higher amounts of IL-6 and IL-8 production compared to fibroblasts infected with planktonic *F. nucleatum* (Figures 3C,D). However, periodontal fibroblasts infected with dispersed *F. nucleatum* produced more cytokines compared to either planktonic or biofilm forms (Figures 3C,D). Although ATP was able to induce dispersal of *F. nucleatum* from biofilm, the magnitude of inflammatory response elicited by ATP-mediated dispersed bacteria did not differ significantly from naturally dispersed *F. nucleatum* (Figures 3E,F). The virulence factors namely, FadA and Fap2 are key bacterial proteins implicated in adhesion and virulence of *F. nucleatum* (Xu et al., 2007; Coppenhagen-Glazer et al., 2015). The expression of *fadA* and *fap2* did not differ significantly between planktonic and biofilm forms of *F. nucleatum*. However, the expression of *fadA* was eight-fold higher in both groups of dispersed *F. nucleatum* while *fap2* expression did not differ significantly between planktonic, biofilm, and dispersed bacteria (Figures 3G,H).

## Discussion

In healthy tissues, ATP concentrations in the extracellular milieu are negligible. While subgingival plaque bacteria is the primary etiological agent of periodontitis, inappropriate host inflammatory responses mounted against plaque bacteria and their products is the main contributor to the progression of periodontal disease (Pihlstrom et al., 2005). A consequence of host tissue damage is the release of intracellular ATP into the extracellular space. Extracellular ATP (eATP) is an example of a danger associated molecular pattern (DAMP) which is employed by multi-cellular organisms to elicit inflammatory and damage healing responses through stimulation of host purinergic receptors (Burnstock et al., 2010). However, the function of eATP appears not to be limited to mammalian cells. Exogenous ATP has been shown to enhance biofilm formation of nosocomial pathogens namely, *E. coli, Acinetobacter baumannii, Stenotrophomonas maltophilia*, and *Staphylococcus aureus* (Xi and Wu, 2010). This occurs through eATP-mediated release of extracellular DNA (eDNA), a key structural component of the EPS of biofilm. However, in this study, we found that ATP did not significantly enhance biofilm formation of *F. nucleatum*. This is likely attributed to the fact that eDNA is not a key component of EPS of *F. nucleatum* biofilm (Ali Mohammed et al., 2013).

Plaque biofilms because of their sessile nature are continuously exposed to various stresses caused by changes in the environment. Although, increased availability of nutrients such as carbon, nitrogen and amino acids and stressors such as nutrient deprivation and oxidative stress have been shown to trigger dispersal of other bacterial species, these factors did not significantly elicit dispersal of *F. nucleatum*. Instead, we found that ATP at concentrations found at sites of inflammation and tissue damage induced dispersal of *F. nucleatum* biofilm. Dispersal, which consists of the coordinated release of single cells from the interior of mature biofilms, likely occurred through ATP's ability to chelate metal ions, depriving the biofilm community of essential metal ions required to maintain the viability of bacteria and the stability of biofilm structure, leading to the release of bacteria from biofilm. Indeed, we showed in this study that supplementation with cations abolished ATP mediated dispersal of *F. nucleatum* biofilm. Induction of dispersal through destabilizing EPS is not a new phenomenon. For instance, *Aggregatibacter actinomycetemcomitans*, a gram negative coccobacilli which is associated with aggressive periodontal disease utilizes the enzyme dispersin B to degrade biofilm matrix, releasing single cells from the biofilm colony (Kaplan et al., 2003).

It is generally assumed that cells dispersed from biofilms immediately go into the planktonic growth phase. However, recent studies showed that biofilm dispersed cells possess phenotypes which are distinct from their biofilm or planktonic counterparts. In particular, dispersed *Candida albicans* are more infectious, displaying 40% increase in both adherence and biofilm forming ability (Uppuluri et al., 2010). Similarly, dispersed *P. aeruginosa* showed significantly increased capacity to kill RAW 264.7 macrophage cells and *Caenorhabditis elegans* (Chua et al., 2014). Regulation of biofilm dispersal in response to host cell damage could be an important mechanism employed by plaque biofilm to enhance their persistence and dissemination. For instance, to have a survival advantage in the oral cavity, bacterial cells released from the biofilm community must adhere to host cells to avoid being washed away by saliva flow. Through transposon library screening of *F. nucleatum* mutants, the virulence factors Fap2 and FadA were found to be important for adhesion of *F. nucleatum* to epithelial cells (Coppenhagen-Glazer et al., 2015). FadA in addition to mediating adhesion to host cells also acts as an invasin, facilitating both adhesion and invasion of host cells via zipper-like mechanisms through rearrangements in cytoskeletal structure (Han et al., 2000). In endothelial cells, it has been elegantly shown that FadA binds to vascular endothelial (VE)-cadherin on endothelial cells causing relocation of VE-cadherin away from cell-cell junctions, consequently increasing endothelial permeability and facilitating dissemination of *F. nucleatum* (Fardini et al., 2011).

We found that both naturally-dispersed and ATP-induced *F. nucleatum* possessed increased adherence and invasion capacity compared to biofilm and planktonic cells where FadA rather than Fap2 was up-regulated in dispersed and not planktonic and biofilm cells. In the absence of exogenous ATP, *F. nucleatum* disperse naturally from biofilm matrix as free planktonic bacteria at low levels. However, both natural and ATP-induced dispersed bacteria did not differ significantly in their adhesive and invasive abilities. Collectively, the data suggest that the enhanced adhesion and invasion of dispersed *F. nucleatum* is likely FadA-mediated.

The increased capacity of dispersed *F. nucleatum* to invade periodontal fibroblasts likely contributed to significantly higher production of pro-inflammatory cytokines in periodontal fibroblasts infected with dispersed *F. nucleatum* compared to their planktonic and biofilm counterparts. Indeed, we have previously shown that the ability of *F. nucleatum* to invade host cells leads to higher pro-inflammatory cytokine production through activation of cytosolic pattern recognition receptors (Lee and Tan, 2014; Quah et al., 2014). Although, periodontal fibroblasts infected with biofilm cells induced significantly more pro-inflammatory cytokine production compared to planktonic cells, this was independent of bacterial invasion since periodontal fibroblasts infected with planktonic of biofilm cells did not differ in their ability to invade. Thus, EPS of *F. nucleatum* could potentially harbor mediators which themselves contribute to pro-inflammatory response in periodontal fibroblasts.

In this study, although extracellular ATP induced dispersal of *F. nucleatum*, bacterial biofilm was cultured on polystyrene surfaces *in vitro* as a mono-species. This differs from physiological conditions in the oral cavity where *F. nucleatum* exists as a consortium of mixed bacterial communities in subgingival plaque biofilm on tooth surfaces bathed in saliva. Therefore, future studies will be required to determine if substratum of *F. nucleatum* biofilm, saliva and oxygen tension could potentially impact extracellular ATP induced dispersal of *F. nucleatum*. In addition, it will be interesting to determine the effect of extracellular ATP on *F. nucleatum* dispersal when present in mixed species biofilm, and also to determine if other metal ion chelators such as EDTA elicit a similar effect on *F. nucleatum* dispersal as extracellular ATP.

Collectively, this study showed for the first time that dispersed periodontal bacteria possess enhanced virulence characteristics. We also showed that periodontal bacterium is capable of co-opting the host signaling molecule eATP to detach from biofilms. A high concentration of eATP potentially indicates depressed host immunity due to cell damage, and an opportune time for bacteria in biofilm to disperse and establish new biofilm colonies, enhancing their colonization, dissemination, and survival. Further studies on the impact of eATP on multispecies periodontal biofilm and virulence will enhance our understanding of the contributory roles of host damage molecules in host-biofilm interactions during periodontal disease.

## Author contributions

QD performed the experiments, analyzed the data and drafted the manuscript. KST conceived the study, analyzed the data, and wrote the manuscript. Both authors have read and approved the final manuscript.

### Conflict of interest statement

The authors declare that the research was conducted in the absence of any commercial or financial relationships that could be construed as a potential conflict of interest.
