# Supplementary figures and images for: The Danger Signal Extracellular ATP Is an Inducer of Fusobacterium nucleatum Biofilm Dispersal
Source: Front Cell Infect Microbiol. 2016 Nov 17;6:155. doi: 10.3389/fcimb.2016.00155 (PMC5112537; doi:10.3389/fcimb.2016.00155)

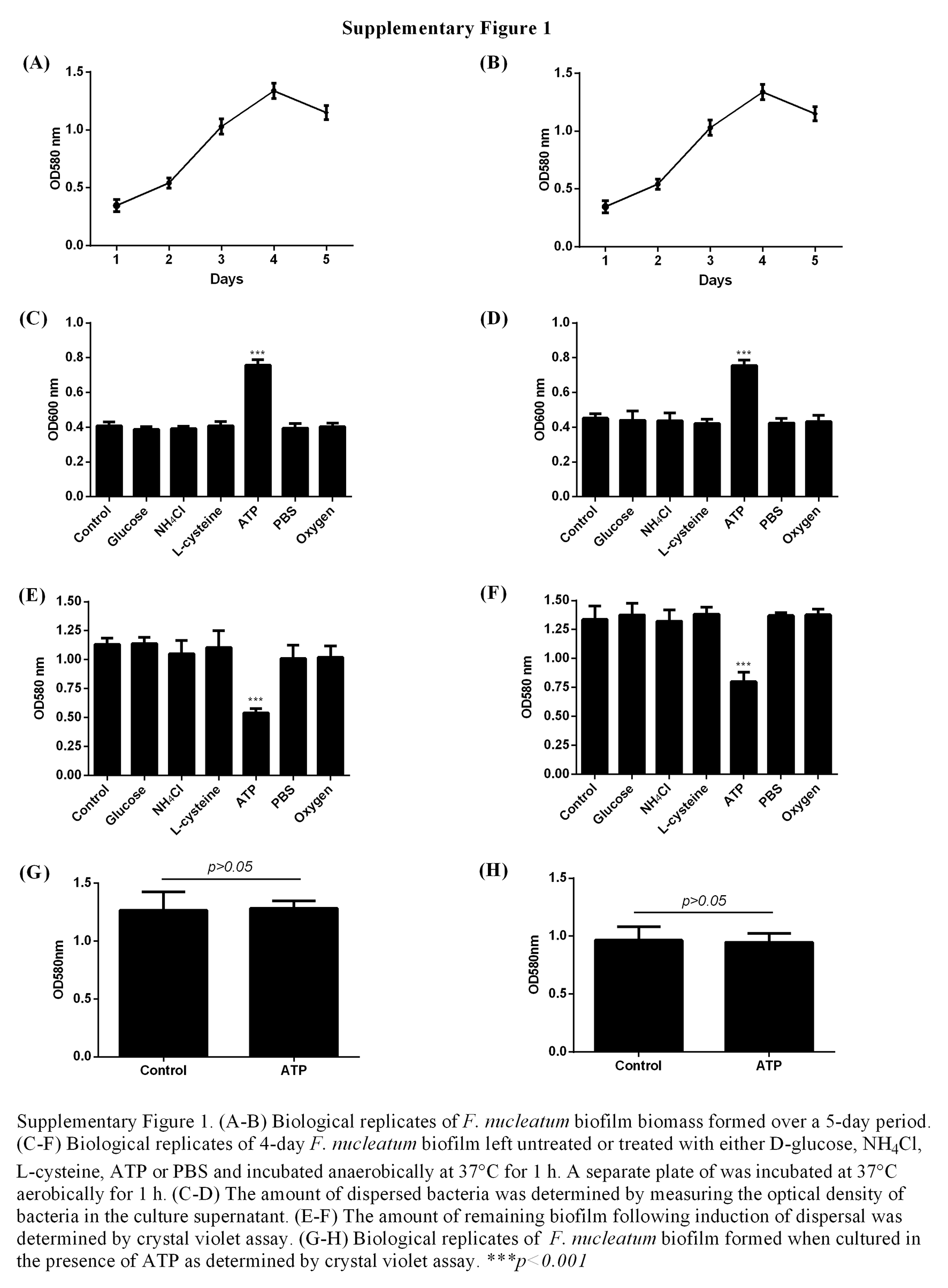

Supplement: Supplementary file 1 [file Image1.TIF]

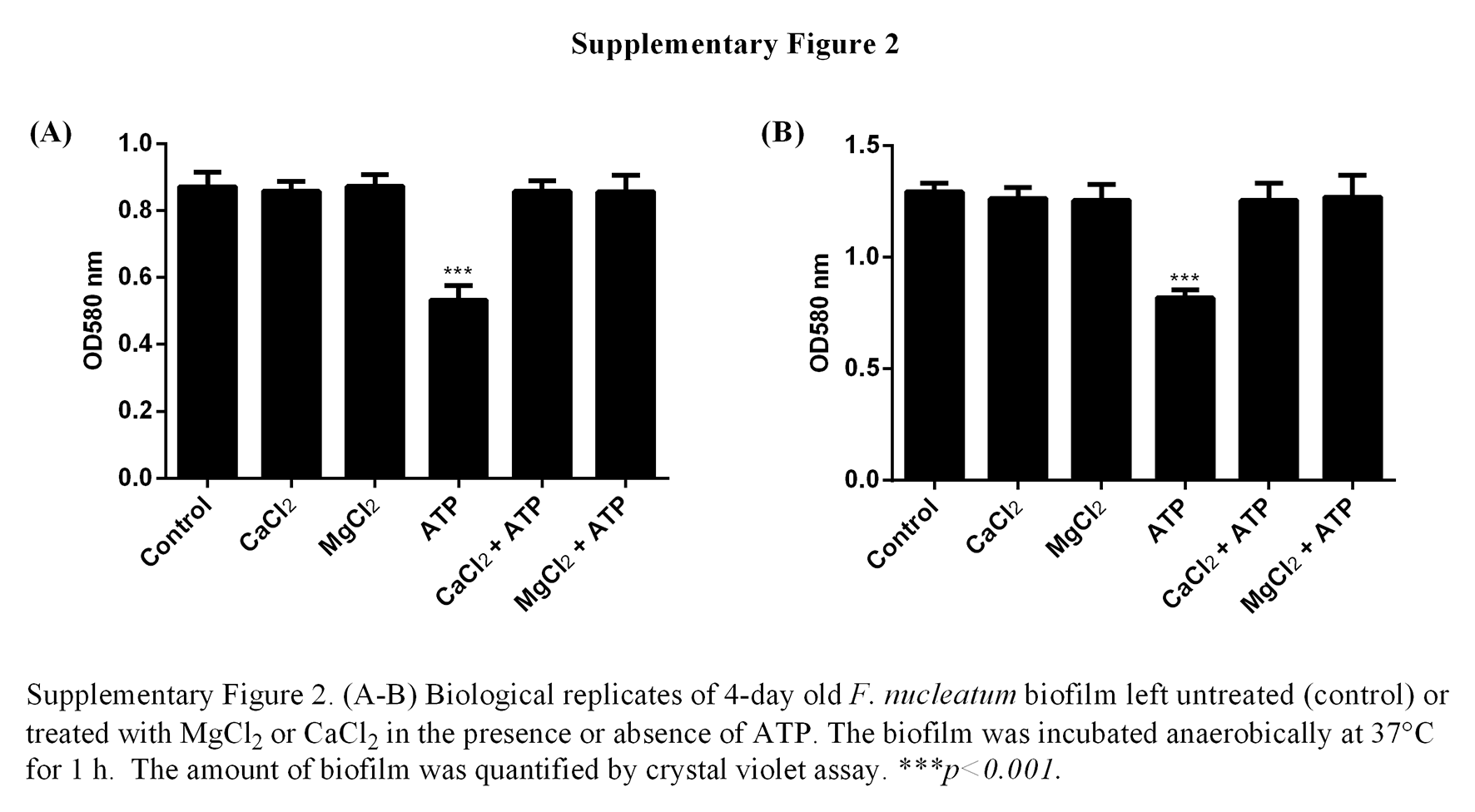

Supplement: Supplementary file 2 [file Image2.TIF]
